# Prophylaxis

**Published:** 1887-02

**Authors:** 


### PROPHYLAXIS.

TRANSLATED FROM BŒNNINGHAUSEN, WITH REMARKS BY A. McNEIL, M. D., SAN FRANCISCO, CALIFORNIA.

"Very nearly related to the subject of antidotes is that of prophylactic remedies, and they are to be judged by the same natural laws. The object of these medicines is to destroy, in advance, in the living organism the disposition to contract a contagious disease. It is known that during the prevalence of even the most malignant and undoubtedly contagious disease there are always some persons unaffected, and the opinion is rational that they are guarded by their want of susceptibility. Experience has shown that in order to produce this condition artificially, only those remedies are efficacious which have the power to cure the disease in question, and to produce a reaction in the organism which is directed against the disease and its influence. Therefore if we will protect the unaffected members of a household in which, for example, a variety of nervous (typhoid) fever exists, we can do it with certainty only by administering that remedy which is truly homœopathic to the symptoms of those who are sick, while all other remedies which may be indicated in nervous fever are entirely useless."—Bœnninghausen's aphorisms of Hippocrates.

I will notice a prevalent error which exists in opposition to the above maxim, viz.: that Belladonna is *the* prophylactic of scarlet fever, and the name of Hahnemann is appealed to in support—we will see with how much truth. See *Materia Medica Pura*, Vol. I, page 97: "Belladonna may be used as a prophylactic against the genuine, erysipelatous, smooth, and glossy scarlet fever, as described by Sydenham, Plencitz, and others. * * * They were ignorant of the character of this disease, which is proper to childhood, and they were indiscreet enough to mistake for scarlet fever the *purple rash*, which had migrated into Germany from Belgium ever since the year 1801. They falsely applied to this purple rash the term 'scarlet fever,' and failed, of course, in trying to cure it by means of the remedy which I had proposed.

"Purple rash (Roonvonk) being a disease different from scarlet fever, it requires to be treated in a different way. In purple rash Belladonna can do no good, and patients who are treated with Belladonna in this will generally have to die."

Hering said scarlatina Sydenhami had not existed in Philadelphia since 1832. I do not think this type of the disease has prevailed in the United States for half a century. The scarlet fever of this generation is the rough or miliary variety. Now let us apply Bœnninghausen's advice to this type of scarlet fever. Where the disease prevails we study our cases as a unit. See Hahnemann's *Organon*, sections 100, 101, and 102. When the epidemic remedy is found it will not only cure the sick, but prevent those exposed to the contagion from contracting the disease.

---

Professor A. E. Small, M. D., of Chicago, formerly of Philadelphia, died in Chicago, December 28th, 1886. We shall probably have an obituary notice of him next month.

